# Feasibility study using multifocal Doppler twinkling artifacts to detect suspicious microcalcifications in ex vivo specimens of breast cancer on US

**DOI:** 10.1038/s41598-022-06939-5

**Published:** 2022-02-21

**Authors:** Vivian Youngjean Park, Jinbum Kang, Kanghee Han, Ilseob Song, Kang-Sik Kim, Se Jin Nam, Ga Ram Kim, Jung Hyun Yoon, Won Seuk Jang, Yangmo Yoo, Min Jung Kim

**Affiliations:** 1 Department of Radiology, Severance Hospital, Research Institute of Radiological Science, Yonsei University College of Medicine, Seoul, 03722 South Korea; 2 Department of Electronic Engineering, Sogang University, Seoul, 04107 South Korea; 3 Department of Bioengineering, University of Washington, Seattle, WA 98105 USA; 4 Department of Health & Medical Equipment, Samsung Electronics Co. Ltd, Suwon, 16678 South Korea; 5 Department of Medical Engineering, Yonsei University College of Medicine, Seoul, 03722 South Korea; 6 Department of Biomedical Engineering, Sogang University, Seoul, 04107 South Korea

**Keywords:** Breast cancer, Cancer imaging

## Abstract

Multifocal Doppler twinkling artifact (MDTA) imaging has shown high detection rates of microcalcifications in phantom studies. We aimed to evaluate its performance in detecting suspicious microcalcifications in comparison with mammography by using ex vivo breast cancer specimens. We prospectively included ten women with breast cancer that presented with calcifications on mammography. Both digital specimen mammography and MDTA imaging were performed for ex vivo breast cancer specimens on the day of surgery. Five breast radiologists marked cells that included suspicious microcalcifications (referred to as ‘positive cell’) on specimen mammographic images using a grid of 5-mm cells. Cells that were marked by at least three readers were considered as ‘consensus-positive’. Matched color Doppler twinkling artifact (CDTA) signals were compared between reconstructed US-MDTA projection images and mammographic images. The median detection rate for each case was 74.7% for positive cells and 96.7% for consensus-positive cells. Of the 10 cases, 90% showed a detection rate of ≥ 80%, with 50% of cases showing a 100% detection rate for consensus-positive cells. The proposed MDTA imaging method showed high performance for detecting suspicious microcalcifications in ex vivo breast cancer specimens, and may be a feasible approach for detecting suspicious breast microcalcifications with US.

## Introduction

Screening mammography is currently the only imaging modality proven to reduce breast cancer-associated mortality, contributing to a 40% reduction in mortality since its introduction in the United States^1^. Such benefits are primarily attributed to its ability to detect early cancer that manifests as calcifications. However, mammography has disadvantages such as radiation and pain related to breast compression, which can lower patient compliance^2, 3^. In addition, its sensitivity substantially decreases as breast density increases, with a sensitivity of about 50% in women with extremely dense breasts^4^.

In contrast, ultrasound (US) can detect small masses regardless of breast density, and causes less or no patient discomfort compared to mammography^3^. Previous trials have consistently shown a significantly higher cancer detection rate with US than mammography alone, with a supplemental yield of approximately 1.7–4.7 per 1000 women^5, 6^. However, primarily due to limitations in detecting calcifications by US, combined mammography and US show higher cancer detection rates than that of both US or mammography alone^5^.

Other studies have investigated various methods to detect calcifications on US, including the use of twinkling artifacts on color Doppler imaging (CDI) and power Doppler imaging^7, 8^. However, it is difficult to consistently present twinkling artifact signals from randomly distributed micron-size calcifications (e.g., breast microcalcifications) with current CDI and as a result, only a few studies have applied CDI to microcalcifications^9, 10^. In a previous report, we developed a new microcalcification detection technique using real-time multifocal Doppler twinkling artifact (MDTA) imaging, and reported comparable detection rates for microcalcifications compared to mammography in phantom studies^11^. In this study, we conducted a reader study using ex vivo breast cancer specimens and aimed to evaluate the performance of real-time MDTA imaging compared to mammography for detecting suspicious microcalcifications.

## Results

### Breast cancer characteristics

Table 1 shows the characteristics of the 10 study participants (median age, 47.5 years; age range 38–69 years) and breast cancers. Of the 10 included cancers, nine were invasive ductal carcinoma and one case was ductal carcinoma in situ.

**Table 1:** Study participants and breast cancer characteristics.

Characteristic	Value
Age (years)	51.1 ± 10.7
**Histologic type**	
Invasive ductal carcinoma	9
Ductal carcinoma in situ	1
**ER/PR status**	
ER or PR positive	5
ER and PR negative	5
**HER2 status**	
HER2 positive	5
HER2 negative	5

Age and lesion size are shown as mean data ± standard deviation.

Unless otherwise indicated, data are number of cancers.

*ER* estrogen receptor, *PR* progesterone receptor, *HER2* = human epidermal growth factor receptor 2.

### Number of positive cells and detection rate

The median number of positive cells by each reviewer per case ranged from 4 to 8.5 cells (see Supplementary Table S1). The average number of positive cells for each case by the five readers ranged from 3.8 to 20.6 cells (Table 2). The average number of matched CDTA signals for each case ranged from 1.6 to 15.6 cells. Therefore, the median detection rate of positive cells for each case was 74.7% (range 38.1–96.4%).

**Table 2:** Detection rate of positive cells for each case.

Case no.	Average No. of positive cells	Average No. of matched CDTA signals	Detection rate of positive cells (%)
1	4.2	3.8	90.5
2	7.6	5.6	73.7
3	4	3.4	85.0
4	5.6	5.4	96.4
5	9.2	8.4	91.3
6	10.8	4.4	40.7
7	20.6	15.6	75.7
8	3.8	2.6	68.4
9	4.2	1.6	38.1
10	5.2	3.4	65.4

### Number of consensus-positive cells and detection rate

The median number of consensus-positive cells, i.e. cells that were marked positive by at least three readers, for each case was 5.5 (range 3–15 cells) (Table 3). The median detection rate of consensus-positive cells for each case was 96.7%, ranging from 50 to 100%. Of the 10 cases, 90% (9 of 10) showed a detection rate ≥ 80%, with 50% (5 of 10) of cases showing a 100% detection rate for consensus positive cells. Only one case showed a detection rate of 50%, which presented as grouped amorphous calcifications on mammography.

**Table 3:** Calcification characteristics and detection rate of consensus-positive cells for each case.

Case no.	Histologic type	Calcification morphology	Calcification distribution	No. of consensus-positive cells	No. of matched CDTA signals	Detection rate of consensus-positive cells (%)
1	IDC	Fine-linear	Grouped	4	4	100
2	IDC	Fine pleomorphic	Segmental	8	7	87.5
3	IDC	Fine pleomorphic	Grouped	5	4	80.0
4	IDC	Fine pleomorphic	Segmental	6	5	83.3
5	IDC	Fine pleomorphic	Segmental	8	8	100
6	IDC	Fine-linear	Segmental	11	11	100
7	IDC	Fine-linear	Regional	15	14	93.3
8	DCIS	Fine pleomorphic	Grouped	3	3	100
9	IDC	Amorphous	Grouped	4	2	50
10	IDC	Coarse heterogeneous	Grouped	4	4	100

*IDC* invasive ductal carcinoma, *DCIS* ductal carcinoma in situ.

## Discussion

Although various studies have suggested that twinkling artifacts on color Doppler and power Doppler imaging can be used effectively to detect calcification (e.g., kidney or urinary stones)^7^, only a few studies have investigated twinkling artifacts in breast microcalcifications^9, 10^. As breast microcalcifications are small in size (< 1 mm) and are more randomly distributed, current color Doppler imaging cannot consistently present twinkling artifacts^11^. Random scattering signals from the rough surfaces of rigid microcalcifications are reflected by MDTA imaging, and the reflected waves constructively and destructively propagate in arbitrary directions and at different angles. In addition, time-varying characteristics that may be caused by phase jitter noise further promote phase fluctuations after clutter filtering, and the arbitrary phase changes are manifested as a rapidly changing pattern on color or power Doppler imaging^12^. By optimizing transmit conditions, we found that the new microcalcification detection technique^11^ showed a high detection rate of suspicious calcifications in breast cancer, with a median detection rate of 96.7% for consensus-positive cells. Our results show that this new technique using MDTA imaging has the potential to overcome the limitations of conventional US by enabling the detection of suspicious microcalcifications in breast cancer.

US is an easily applicable imaging tool and currently the most widely used imaging modality in both supplemental breast cancer screening and diagnostic breast imaging. Unlike mammography, US requires less breast compression and no radiation exposure, which decreases patient discomfort and allows its application to even pregnant or very young patients^13^. While US-guided breast biopsy is preferred over mammography-guided stereotactic biopsy^14, 15^, stereotactic biopsy is still widely performed due to the inferior performance of US for detecting suspicious microcalcifications. However, approximately 2% of stereotactic biopsy procedures fail due to technical reasons, including difficulties in lesion location or thin breasts which may be accessible with US-guidance^16, 17^. The MDTA imaging method may be useful when US-guided biopsy is performed for suspicious microcalcifications, by improving both patient comfort and reducing unnecessary excisional biopsies.

Although the assessment of breast microcalcifications is based on morphology and distribution on mammography, the interobserver agreement for calcifications is lower than the other BI-RADS lexicon descriptors^18, 19^. Therefore, when using radiologists’ assessments, further consensus reading and the combination of results from multiple readers are imperative to establishing a reliable reference standard. In our study, the median number of positive cells (i.e., cells considered to include suspicious calcifications) counted by each reviewer per case ranged from 4 to 8.5, also implying interobserver variability. It is notable that even though the detection rate for all positive cells (i.e., marked by at least one reader) was 40.7% in one case, the detection rate of consensus-positive cells (i.e., marked by at least three readers) was 100%. Therefore, although the MDTA imaging method may not detect all calcifications, its higher performance in detecting the most significant microcalcifications will be helpful when guiding biopsy.

However, despite the small number of cases, we found that the MDTA imaging method may have less additional value when detecting amorphous calcifications, which have been reported to be difficult to detect with B-mode US as well^20^. The single case that showed a detection rate lower than 80% for consensus-positive cells presented as amorphous grouped microcalcifications on mammography, with a detection rate of 50% using MDTA imaging. Further studies are needed to determine which type of suspicious microcalcifications will benefit the most from MDTA imaging.

Our study has a number of limitations. First, the number of included cases was small. Second, as only breast cancer cases were included, we were just able to investigate the detectability of malignant microcalcifications. Although such detection rates have been used in previous feasibility studies that compared new techniques to conventional imaging, we were unable to provide detailed diagnostic performance measures. However, a high performance in detecting malignant microcalcifications is useful for image-guided biopsy, especially when choosing target areas. Third, as this was a pilot study using ex vivo breast cancer specimens, we were unable to evaluate effects from blood flow signals, which would also generate Doppler signals. However, the unique signal characteristics of calcifications compared to those of blood flow signals (i.e., no dependency on the pulse repetition frequency, high variance of the Doppler phase, and no pulsation) will help in discriminating and eliminating the blood flow signals in vivo^11^.

In conclusion, US using a MDTA imaging technique showed a high detection rate of 96.7% for suspicious microcalcifications in ex vivo breast cancer specimens. The proposed MDTA imaging method provided another feasible approach for detecting suspicious breast microcalcifications on US.

## Methods

### Study participants

**Figure 1:**
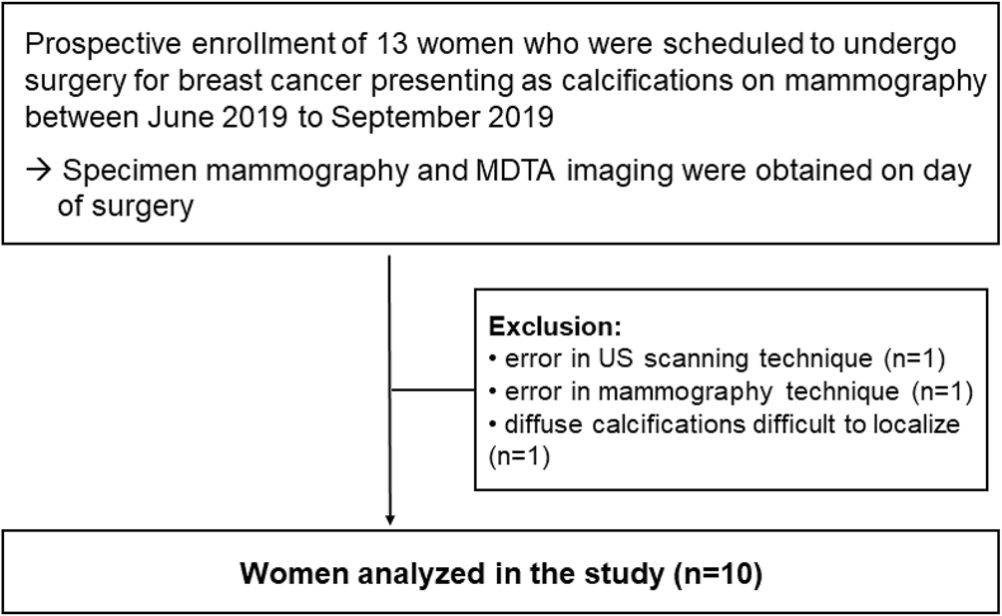
Flowchart of study participants.

This prospective study was approved by the institutional review board of Severance Hospital (IRB number: 1-2019-0014), and all participants provided written informed consent. All experiments were performed in accordance with relevant guidelines and regulations. From June 2019 to September 2019, 13 women who were scheduled to undergo surgery for breast cancer that presented as calcifications on mammography were enrolled, with specimen mammography and specimen US-MDTA imaging being performed on the day of surgery. Of the 13 patients, three were excluded due to errors in the US scanning technique (n = 1) or mammography (n = 1), and due to diffuse calcification involving the breast for which localization was difficult (n = 1). Finally, 10 women with malignant breast calcifications were included (Fig. 1).

### Specimen mammography

Immediately following surgical removal of the tumor, surgical specimens of the tumor were carried to the mammography unit and specimen mammography was performed using a full-field digital mammography system (Selenia Dimensions; Hologic, Bedford, MA). First, the surgical specimen was positioned on the mammography plate and a digital magnification view was obtained in the top-down image plane, with the specimen slightly flattened with the compression paddle to equalize the tissue in a magnification view of 1:1.8. This magnification view was obtained only to aid the readers in the assessment of calcifications.

Next, the surgical specimen was placed inside a sterilized acrylic tank (30 × 25 × 12 cm) and held by a sterilized mesh to prevent movement during scanning. The tank was then positioned on the mammography plate and digital specimen mammography was performed. These mammographic images were correlated with the US-MDTA images for image review.

### Multifocal Doppler twinkling artifact (MDTA) imaging

After mammographic images were acquired, the tank was filled with cold saline, and 3D mechanical scanning was performed with real-time MDTA imaging using an ultrasound research platform (Vantage 128, Versonics Inc., Redmond, WA, USA) while moving a linear array transducer (L11-5v, Versonics Inc., Redmond, WA, USA) with motion stage controller (see Supplementary Fig. S1)^11^. During scanning, cross-sectional ultrasound data were captured with the ultrasound research platform while the transducer was moving, and the instantaneous motion vector was simultaneously obtained by a commercial motion tracking system (TrakSTAR, Ascension Tech. Corp., Shelburne, VT, USA) with a magnetic motion sensor attached to the transducer. For real-time US-MDTA imaging, three focus beams with optimized acoustic parameters (e.g., the number of focusing, focal depth and f-number) were employed to maintain the proper frame rate during mechanical scanning. The MDTA imaging with the optimized transmit conditions highly increased the sensitivity of TA signals after clutter filtering, and all higher TA signals than background noise level were visualized and considered as microcalcifications, as described in our previous reports^21^.

The acquired 2D images were subsequently reconstructed to a volumetric image by a common voxel-based algorithm. To directly compare US-based MDTA images with the transverse mammographic images, a projection image along the z-direction (C-plane) was produced by the volumetric image and a spatial peak detection from the projected MDTA image was consecutively performed for better visual assessment.

### Assessment of ex vivo specimens

Specimen mammographic images were independently reviewed by five board-certified radiologists with 6, 7, 7, 11, and 18 years of subspeciality experience in breast imaging, respectively. A grid composed of 5-mm cells was superimposed on a PACS monitor, and each reader reviewed the specimen mammographic images and marked all cells that included suspicious microcalcifications (henceforth referred to as ‘positive cells’) (see Supplementary Fig. S2). The readers were allowed to refer to the specimen magnification mammographic images when assessing calcification morphology.

**Figure 2:**
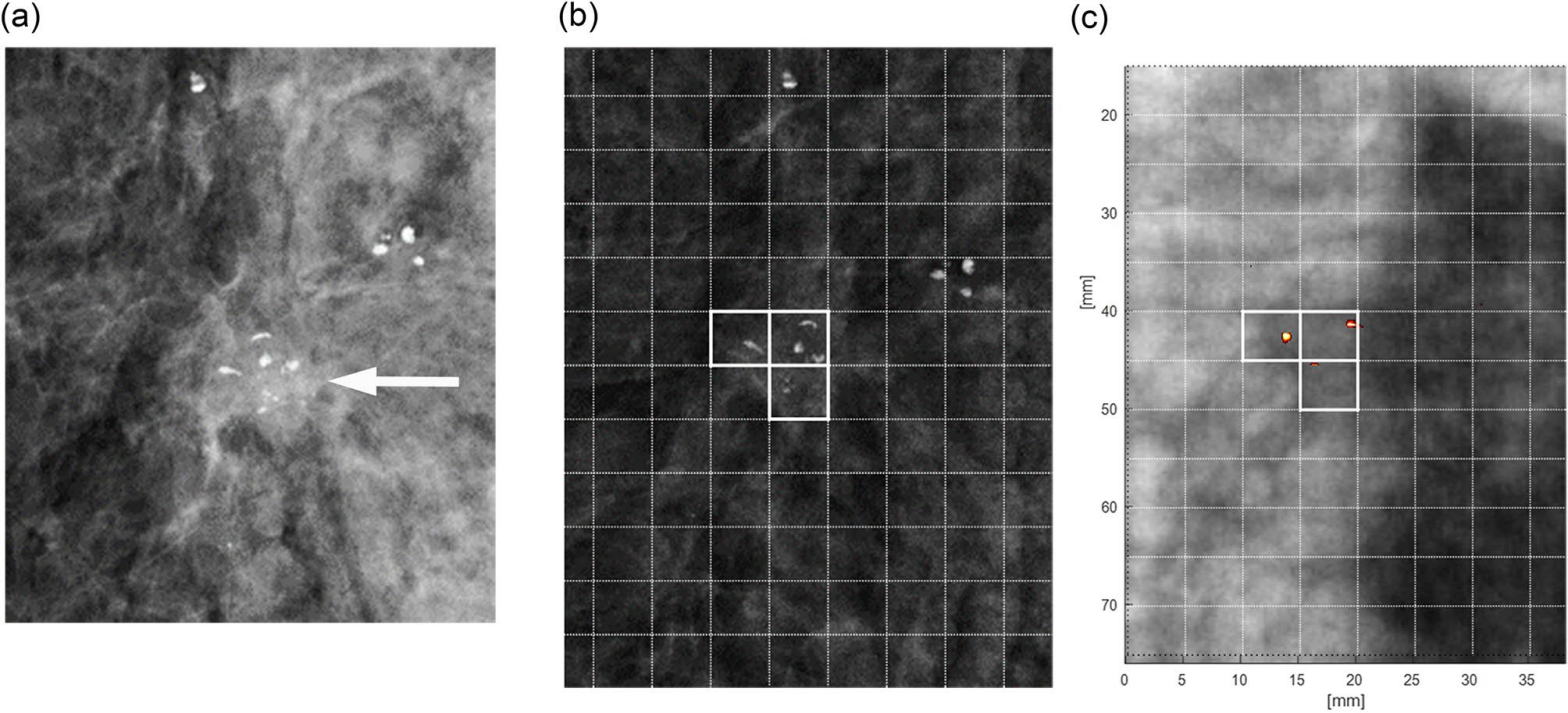
Mammographic and color-encoded US-multifocal Doppler twinkling artifact (MDTA) projection images of an invasive ductal carcinoma in a 64-year-old woman. **(a)** Specimen magnification mammographic image shows the malignancy as a mass with internal calcifications (arrow). Other diffuse calcifications at the right breast were assessed as benign. **(b)** Consensus-positive cells are shown on the specimen mammographic image (cells outlined in bold). These were marked by using a grid composed of 5-mm cells. **(c)** A color-encoded 2D projection image along the z-axis using the US-MDTA volume data showed a 100% detection rate for the consensus-positive cells.

Subsequently, the location of each cell was converted to numbers by row and column, and any cells that were marked were recorded for each case. Cells that were marked by at least three readers were considered as ‘consensus-positive’. Positive and consensus-positive cells were then compared with color-encoded US-MDTA projection images along the z-direction (C-plane) using spatial peak detection (Fig. 2).

Two radiologists (V.Y.P and M.J.K., with 8 and 20 years of subspeciality experience in breast imaging), reviewed preoperative mammographic images in consensus and assessed calcification morphology and distribution based on the American College of Radiology Breast Imaging Reporting and Data System (BI-RADS) lexicon^22^.

### Statistical analysis

The number of positive cells were calculated for each case and each reader, and the number of consensus-positive cells were calculated for each case. A matched color Doppler twinkling artifact (CDTA) signal was defined as a CDTA signal detected on US-MDTA projection images corresponding to the spatial location of the cell marked as positive (i.e., containing suspicious microcalcifications). We then calculated the detection rate of positive cells per case, which was calculated by dividing the sum of positive cells by the total number of matched CDTA signals from all five readers. We also calculated the detection rate of consensus-positive cells per case, which was calculated by dividing the sum of consensus positive cells by the total number of matched CDTA signals from all five readers.

## Supplementary Information

Supplementary Information.

## Data Availability

The datasets used and/or analysed during the current study are available from the corresponding author on reasonable request.
