# Supplementary material for: Feasibility study using multifocal Doppler twinkling artifacts to detect suspicious microcalcifications in ex vivo specimens of breast cancer on US
Source: Sci Rep. 2022 Feb 21;12:2857. doi: 10.1038/s41598-022-06939-5 (PMC8861000; doi:10.1038/s41598-022-06939-5)
Supplement: Supplementary file 1 — Supplementary Information. [file 41598_2022_6939_MOESM1_ESM.pdf]

Supplementary Figure

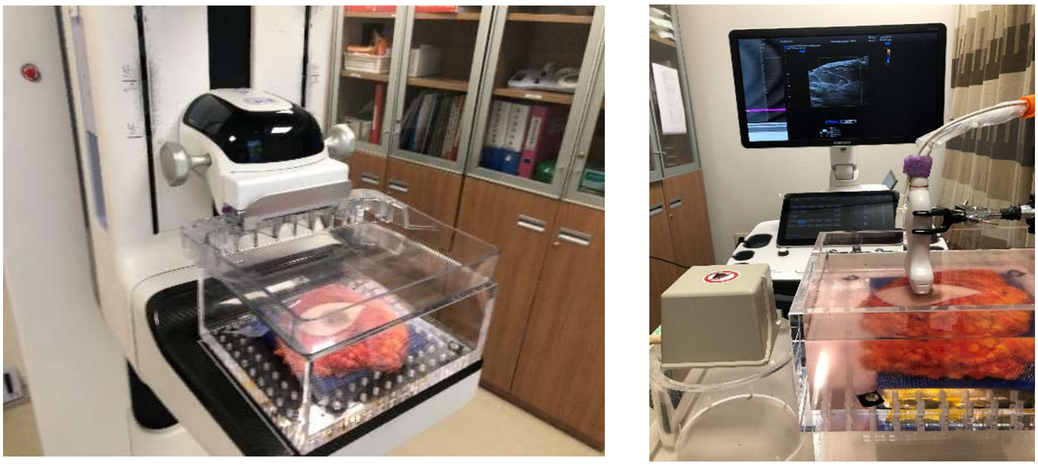

(a). **(b).**

Figure S1. Specimen mammography and ex vivo multifocal Doppler twinkling artifact (MDTA) imaging of surgical specimens. **(a)** Breast surgical specimens were placed inside a sterilized acrylic tank and held by a sterilized mesh. Digital specimen mammography was then performed after positioning the tank on the mammography plate. **(b)** The tank was then filled with saline, and 3D mechanical scanning was performed with real-time MDTA imaging using an ultrasound research platform (Vantage 128, Versonics Inc., Redmond, WA, USA).

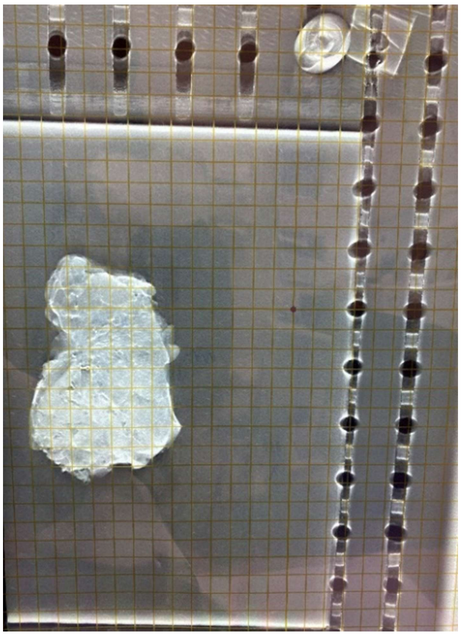

Figure S2. Position of grid on a PACS monitor. A grid composed of 5-mm cells was superimposed on a PACS monitor, and each reader reviewed the specimen mammographic images and marked all cells including suspicious microcalcifications (positive cells). Subsequently, the location for each cell was converted to numbers based on its location by row and column, and cells that were marked were recorded for each case. for location correlation between specimen mammographic images and US-MDTA projection images.

Table S1. Number of positive cells according to reviewer.

Reviewer	Median No. of positive cells by reviewer (mean, minimum, maximum)
1	4<br>(6.6, 3, 14)
2	4.5<br>(5.1, 2, 10)
3	5<br>(8.1, 3, 25)
4	6<br>(11.1, 3, 38)
5	8.5<br>(9.9, 4, 20)
